# The development of bronchiectasis on chest computed tomography in children with cystic fibrosis: can pre-stages be identified?

**DOI:** 10.1007/s00330-016-4329-z

**Published:** 2016-04-23

**Authors:** Leonie A. Tepper, Daan Caudri, Adria Perez Rovira, Harm A. W. M. Tiddens, Marleen de Bruijne

**Affiliations:** 1 Department of Pediatric Pulmonology, Erasmus MC, Sophia Children’s Hospital, Rotterdam, The Netherlands; 2 Department of Radiology, Erasmus MC, Sophia Children’s Hospital, Rotterdam, The Netherlands; 3 Biomedical Imaging Group Rotterdam, Departments of Radiology and Medical Informatics, Erasmus MC, Rotterdam, The Netherlands; 4 Department of Pediatric Pulmonology and Radiology, Erasmus Medical Center, Sophia Children’s Hospital, Dr. Molewaterplein 60, room SP-3464, 3015 GJ Rotterdam, The Netherlands; 5 Department of Computer Science, University of Copenhagen, Copenhagen, Denmark

**Keywords:** Cystic fibrosis, Paediatrics, High resolution computed tomography, Bronchiectasis, Lung disease

## Abstract

**Objective:**

Bronchiectasis is an important component of cystic fibrosis (CF) lung disease but little is known about its development. We aimed to study the development of bronchiectasis and identify determinants for rapid progression of bronchiectasis on chest CT.

**Methods:**

Forty-three patients with CF with at least four consecutive biennial volumetric CTs were included. Areas with bronchiectasis on the most recent CT were marked as regions of interest (ROIs). These ROIs were generated on all preceding CTs using deformable image registration. Observers indicated whether: bronchiectasis, mucus plugging, airway wall thickening, atelectasis/consolidation or normal airways were present in the ROIs.

**Results:**

We identified 362 ROIs on the most recent CT. In 187 (51.7 %) ROIs bronchiectasis was present on all preceding CTs, while 175 ROIs showed development of bronchiectasis. In 139/175 (79.4 %) no pre-stages of bronchiectasis were identified. In 36/175 (20.6 %) bronchiectatic airways the following pre-stages were identified: mucus plugging (17.7 %), airway wall thickening (1.7 %) or atelectasis/consolidation (1.1 %). Pancreatic insufficiency was more prevalent in the rapid progressors compared to the slow progressors (p = 0.05).

**Conclusion:**

Most bronchiectatic airways developed within 2 years without visible pre-stages, underlining the treacherous nature of CF lung disease. Mucus plugging was the most frequent pre-stage.

***Key Points*:**

*• Development of bronchiectasis in cystic fibrosis lung disease on CT.*

*• Most bronchiectatic airways developed within 2 years without pre-stages.*

*• The most frequently identified pre-stage was mucus plugging.*

*• This study underlines the treacherous nature of CF lung disease.*

**Electronic supplementary material:**

The online version of this article (doi:10.1007/s00330-016-4329-z) contains supplementary material, which is available to authorized users.

## Introduction

An important component of cystic fibrosis (CF) lung disease is bronchiectasis [1]. Bronchiectasis is a permanent and irreversible abnormal dilatation of the bronchial lumen and is most reliably detected by chest computed tomography (CT) [2–5].

Bronchiectasis has been observed on chest CT in infants with CF as young as 10 weeks of age [6–11]. At 3–5 years of age, 50–70 % of children with CF already have bronchiectasis, which contributes importantly to the morbidity and mortality in CF [6, 8, 12]. To prevent the development of bronchiectasis it is of great clinical importance to identify early pre-stages on CT with the aim of preventing further progression. Mucus plugging, airway wall thickening and atelectasis or consolidation have been observed to develop early in life in many patients and these changes could potentially be pre-stages for the development of bronchiectasis [1]. Studies showed that once bronchiectatic airways are present, they progress in severity [1, 6, 10, 13, 14]. However, it remains unclear how bronchiectatic airways evolve and why progression is more rapid in some patients [15].

Therefore, this study aims to identify pre-stages of bronchiectasis in CF on chest CT and to determine which patients are at risk for a rapid progression of bronchiectasis.

## Methods

### Study population

This retrospective study used longitudinally collected clinical data from the routine annual evaluation of CF patients under treatment in the Erasmus MC-CF Center (Rotterdam, The Netherlands) from January 2005–May 2013. We included 43 clinically stable CF patients who had at least four consecutive volumetric inspiratory CTs to guarantee a minimum follow-up period of 6 years. A routine chest CT is performed biennially as part of the annual evaluation programme. During this study period the annual evaluation programme was changed to structure the follow-up schedule for routine chest CTs, so that every child with CF has a CT at the same age (6, 8, 10, 12, 14, 16, 18 years).

Patients who had a lung transplant in the study period were excluded. Furthermore, patients with a pulmonary exacerbation or pulmonary complications (e.g. pneumothorax or haemoptysis) at the time of CT scanning were excluded. A pulmonary exacerbation was defined as receiving intravenous antibiotics for respiratory symptoms.

We denoted the most recent available volumetric CT made during annual evaluation as CT_baseline_. CT_minus2,4,6,8_ are denoted as respectively the CTs made 2, 4, 6 or 8 years prior to CT_baseline_.

This study was approved by the Institutional Review Board of the Erasmus MC-CF Center (MEC-2013-593).

### CT acquisition and spirometry

CTs were performed on different CT scanners using different low-dose protocols. Most CTs (77.8 %) were predominantly executed on the Emotion 6 (Siemens Emotion 6, Siemens Healthcare, Germany). Since 2010 the remainder of the scans were made on a new CT scanner (SOMATOM Definition Flash, Siemens Healthcare, Germany). More details about the CT protocols and CT scanners are given in the on-line supplementary material (e-Table 1).

From 2007 onwards, most volumetric CTs were spirometer controlled. Spirometer-controlled CT scanning was introduced in our hospital to optimize inspiratory and expiratory volume. If due to logistic reasons CT was not spirometer controlled, then training for breath holds prior to the scan and instructions during the scan were given by the lung function technician (on-line supplementary material).

Spirometry was performed at the annual evaluation using a diagnostic system (Jaeger AG, Würzburg, Germany). The spirometry parameters included for analysis were forced expiratory volume in 1 s (FEV_1_) and forced vital capacity (FVC). FEV_1_ and FVC were expressed as a percentage of predictive values, calculated using the Stanojevic et al. reference equations [16].

### CT analysis

**Fig. 1:**
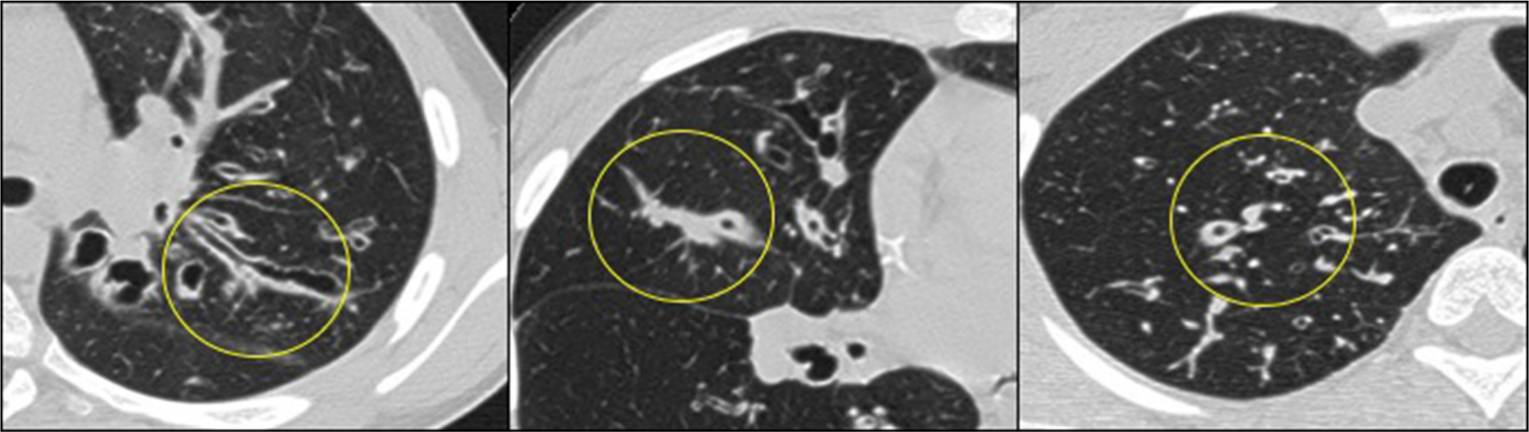
Illustration of scoring categories: bronchiectasis (left), mucus plugging (middle) and airway wall thickening (right)

**Fig. 2:**
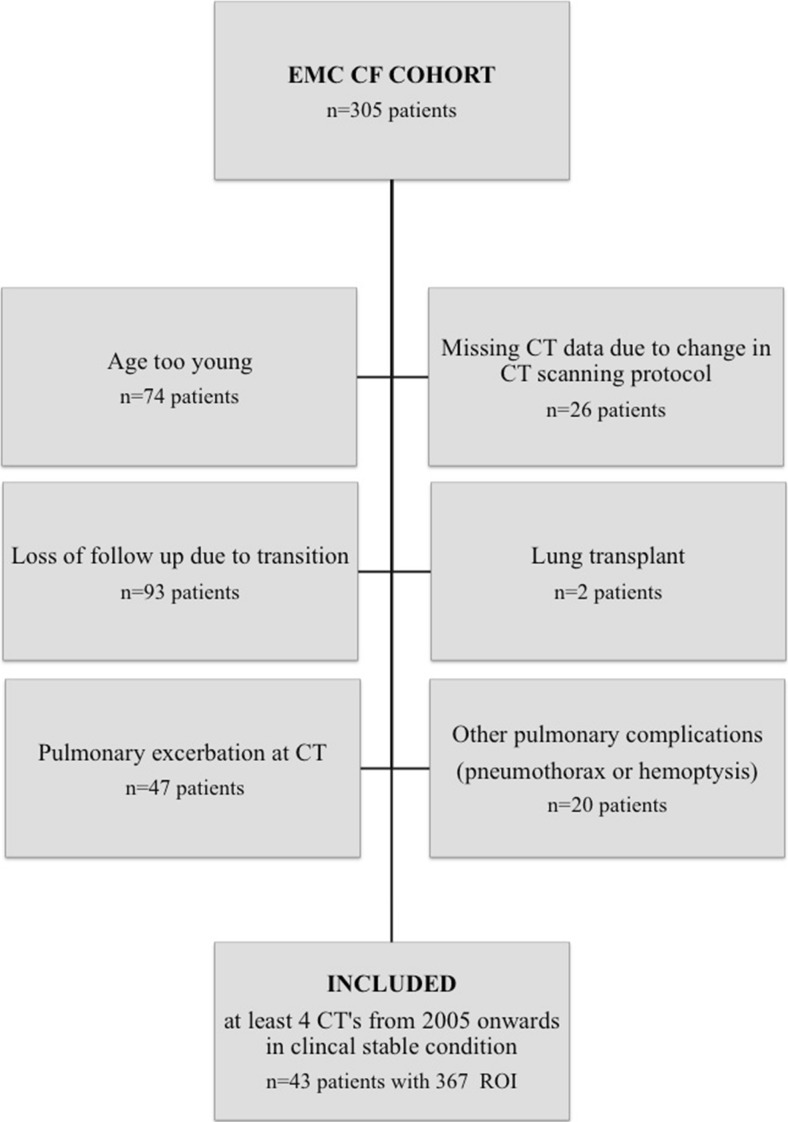
Flowchart of the study population. *CF* cystic fibrosis, *ROIs* regions of interest

**Fig. 3:**
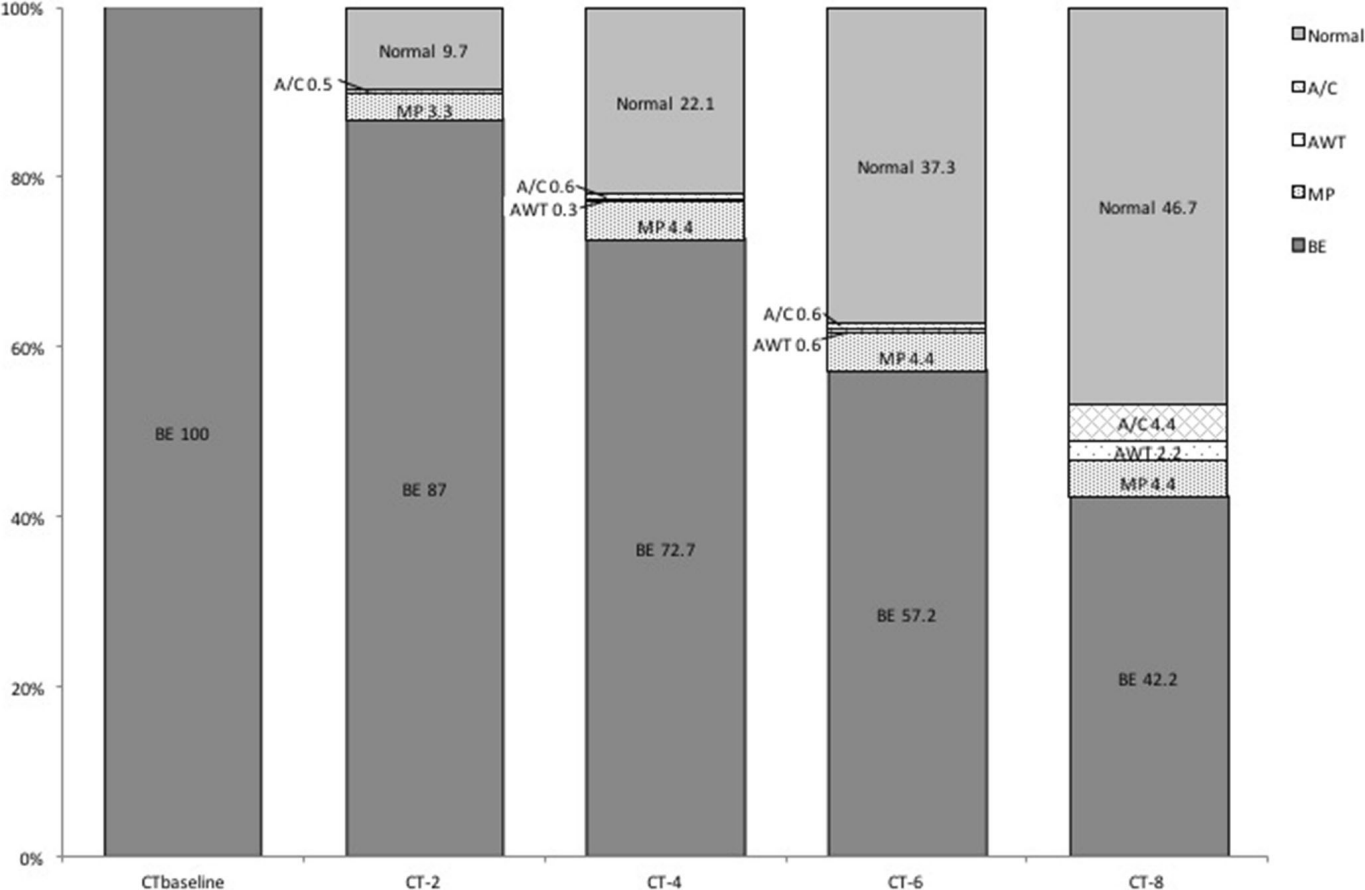
Structural lung damage over time (n = 362 in 43 patients). Dark grey represents bronchiectasis, light-dark grey (area above dark grey) represents mucus plugging, grey dots represent airway wall thickening, white dots represent atelectasis/consolidation, light grey (on top) represents normal airways. CT_baseline_ is the most recent CT

According to the definition of the validated CF-CT scoring system [17–19], bronchiectasis is present if the bronchial lumen diameter is larger than the adjacent pulmonary artery outer diameter, or if there is a lack of tapering for at least 2 cm distal to a branching point. Based on literature about the pathophysiology of bronchiectasis and based on the expertise of a panel consisting of a paediatric pulmonologist (HT), a radiologist (PC), a biomedical imaging expert (MB) and a PhD student (LT), five mutually exclusive categories for classifying pre-stages of bronchiectasis were defined. These categories were: (1) bronchiectasis (bronchial lumen diameter is larger than the adjacent pulmonary artery outer diameter, or lack of tapering for at least 2 cm, Fig. 1), (2) mucus plugging (filling of clearly identifiable bronchi, Fig. 1), (3) airway wall thickening (ratio between the bronchial wall thickness and the outer diameter of the adjacent pulmonary artery being more than 33 %, Fig. 1), (4) atelectasis or consolidation and (5) normal airways. These categories are well defined as part of the CF-CT scoring system and are further explained in the on-line supplementary material [17–19].

CTs were de-identified and randomized. One observer (observer 2: DZ) encircled all areas with bronchiectasis (bronchial lumen diameter-pulmonary artery outer diameter ratio > 2 or saccular bronchiectasis) in the most recent volumetric inspiratory CT (CT_baseline_). Those areas were marked as a region of interest (ROI) using tools in our image analysis platform (®Myrian Onco XL, Intrasense, France). By using deformable image registration, the same areas were automatically identified in the previous CTs (CT_minus2-minus8_). All preceding ROIs were assessed by two experienced observers with respectively 1 and 2 years of experience (HO and DZ) and assigned to one of the five above-mentioned categories. Category 2–5 reflects a potential pre-stage in the ROI of the bronchiectatic airway observed on the most recent CT.

ROIs were assessed in random order with respect to patients and order of CT scans (CT_minus2-minus8_). Therefore, the observers had no knowledge of the assigned category of the ROIs in previous or later scans.

### Statistical analysis

To assess observer agreement kappa scores were calculated. For the interobserver agreement, a random subset of 35 patients with a total of 1,230 ROIs were scored by two observers (H.O. and D.Z.). For the intra-observer agreement, a subset of 20 patients with 195 ROIs were rescored by H.O. after 1 month. In the final analyses, only the scores of observer H.O. were used. Although no universally accepted standards are available for what constitutes good agreement, kappa scores of < 0.40, between 0.4 and 0.75, and ≥ 0.75 are considered to represent poor, moderate to good and excellent agreement, respectively [20].

Descriptive statistics were used to describe the CT findings.

We calculated how often bronchiectasis was persistent in all CTs and how often it was directly preceded by mucus plugging, airway wall thickening, atelectasis/consolidation, or normal airways.

In order to distinguish patients who rapidly developed bronchiectasis from previously normal airways from the ones who were likely to have a pre-stage, we created two progression groups: a rapid progression group and a slow progression group. The number of ROIs in which bronchiectasis was directly preceded by normal airways in any two successive scans was calculated. The median number of these rapidly progressing ROIs per patient was calculated (median = 3) and used as the cut-off point to define the groups of rapid and slow progressors. Patients who had more than three ROIs in which normal airways became bronchiectatic within 2 years were thus included into the rapid progression group (n = 21); the others were included in the slow progression group (n = 18). The two groups were compared with respect to baseline characteristics using Chi-square tests.

SPSS version 13.0 was used for the analyses in this study. Values are shown as median (range) unless otherwise indicated. P-values < 0.05 (two-tailed) are considered to be statistically significant.

## Results

### Study population and CT data

Forty-three patients (18 males) with a median age of 15.3 years (range 9–24 years) were included (Fig. 2). Of those 43 patients, 39 patients had four consecutive CTs and four patients had five consecutive CTs. The median interval between two CTs was 2.0 years (range 0.8–3.9). Baseline characteristics of the study cohort are shown in Table 1.

**Table 1:** Baseline characteristics of the study cohort

Characteristic	CT_baseline_	CT_minus 2_	CT_minus 4_	CT_minus 6_	CT_minus 8_
Number of patients	43	43	43	43	4
Number of regions of interest	367	367	367	367	46
Gender (males)	18 (41.9)	18 (41.9)	18 (41.9)	18 (41.9)	3 (75.0)
Age, year	15.3 (9–24)	13.2 (6–22)	11.0 (4–18)	9.0 (2–16)	6.8 (2–10)
SES^#^					
Low	12 (29.3)	12 (29.3)	12 (29.3)	12 (29.3)	1 (25.0)
Average	15 (36.6)	15 (36.6)	15 (36.6)	15 (36.6)	1 (25.0)
High	13 (31.7)	13 (31.7)	13 (31.7)	13 (31.7)	2 (50.0)
Scientific	1 (2.4)	1 (2.4)	1 (2.4)	1 (2.4)	0 (0.0)
Genetics					
Homozygous dF508	30 (69.8)	30 (69.8)	30 (69.8)	30 (69.8)	3 (75.0)
Heterozygous dF508	9 (20.9)	9 (20.9)	9 (20.9)	9 (20.9)	1 (25.0)
Heterozygous other mutation	4 (9.3)	4 (9.3)	4 (9.3)	4 (9.3)	0 (0.0)
Presence of co-morbidities^‡^					
Pancreatic insufficient	40 (93.0)	40 (93.0)	40 (93.0)	40 (93.0)	3 (75.0)
CFRD	10 (23.3)	10 (23.3)	10 (23.3)	10 (23.3)	0 (0.0)
Asthma	3 (7.0)	3 (7.0)	3 (7.0)	3 (7.0)	0 (0.0)
ABPA	2 (4.7)	2 (4.7)	2 (4.7)	2 (4.7)	0 (0.0)
Chronic colonization *Pa* ^‡‡^	8 (18.6)	8 (18.6)	8 (18.6)	8 (18.6)	1 (25.0)
BMI	19.2 (15–36)	18.0 (14–30)	17.0 (5–31)	16.5 (14–25)	15.7 (15–17)
FEV_1_, % predicted	86.5 (33–106)	85.4 (37–112)	85.0 (40–113)	85.8 (42–126)	88.4 (85–92)
FVC, % predicted	94.0 (49–115)	92.3 (56–128)	95.2 (57–118)	93.2 (49–128)	99.0 (94–102)

Data are presented as no. (%) or median (range)

CT_baseline–_ _minus8_ indicate the time points at which the CTs were made, with CT_baseline_ representing the most recent CT and CT_minus2-8_ respectively representing the CT made 2, 4, 6 or 8 years before CT_baseline_

^#^ SES: socio-economic status based on the highest level of education of either parent (n = 41 at CT_baseline–_ _minus6_ and n = 4 at CT_minus__8_)

^‡^ Indicating the number of patients having co-morbidities

^‡ ‡^ Chronic colonization *Pa*: defined as ≥ three consecutive positive respiratory cultures for *Pseudomonas aeruginosa* (*Pa*) from birth to CT_baseline_

*CFRD* CF-related diabetes, *ABPA* allergic bronchopulmonary aspergillosis, *BMI* body mass index, *FEV*_*1*_ forced expiratory volume in 1 s, *FVC* forced vital capacity

In the CT_baseline_ of the included 43 patients, 367 unique ROIs were identified. In 5/367 (1.4 %) ROIs, bronchiectasis turned into a different category before being scored as bronchiectasis in the baseline scan. In three of the five ROIs the following pathway was identified: bronchiectasis in which the CT immediately preceding the CT with bronchiectasis showed normal airways, mucus plugging, bronchiectasis and normal airways. In two of the five ROIs the following pathway was identified: bronchiectasis in which the CT immediately preceding the CT with bronchiectasis showed atelectasis/consolidation, bronchiectasisand normal airways.

Previous literature showed that bronchiectasis is irreversible and therefore we excluded those 5/367 cases from further analysis. An overview of the structural lung damage (CT_baseline_-CT_minus8_) in our study period is shown in Fig. 3.

The kappa for intra-observer agreement was 0.77 and for interobserver agreement 0.48. More detailed information about the intra- and interobserver agreement is displayed in Table 1 in the on-line supplementary material.

### Bronchiectasis and pre-stages (Fig. 3)

In 187 of the 362 (51.7 %) ROIs bronchiectasis was persistent in all available CTs. For the remaining 175 ROIs in which development of bronchiectasis could be observed, the CT immediately preceding the first CT with bronchiectasis showed normal airways (139/175, 79.4 %), mucus plugging (31/175, 17.7 %), airway wall thickening (3/175, 1.7 %) or atelectasis/consolidation (2/175, 1.1 %).

The most commonly identified pre-stage of bronchiectasis was mucus plugging. In 23 ROIs mucus plugging turned into bronchiectasis within one CT (2 years, range 1.7–2.4). In the other eight ROIs mucus plugging persisted for two CTs (4 years, range 3.7–5.9) before progression to bronchiectasis. Mucus plugging never remained longer than three consecutive CTs, without progressing to bronchiectasis.

### Progression to bronchiectasis

Based on the median number of three ROIs per patient that progressed from normal to bronchiectasis in 2 years, we included 21 patients in the rapid progression group and 18 patients in the slow progression group. There was no statistical difference between these groups with regard to gender (p = 0.47), socioeconomic status (p = 0.49), genetic defect (p = 0.78), CF-related diabetes (CFRD, p = 0.16), asthma (p = 0.12), allergic bronchopulmonary aspergillosis (ABPA, p = 0.12) or chronic *Pseudomonas aeruginosa* infection (p = 0.39). However, there was a borderline significant difference (p = 0.05) for pancreatic insufficiency: all patients included in the rapid group were pancreatic insufficient, while 15 of the 18 patients in the slow progression group were pancreatic insufficient.

## Discussion

This is the first longitudinal study of CT-diagnosed bronchiectasis aiming to identify CT detectable pre-stages of bronchiectasis in children with CF. We assessed the evolution of uniquely identified ROIs using a deformable image registration technique. Our observations provide a unique insight into the radiological course of developing bronchiectasis.

Our most important findings are that most bronchiectasis appeared within the 2-year timeframe between successive scans. Of the pre-stages that could be identified, mucus plugging was the most common predecessor of bronchiectasis.

Mucus plugging was identified as a potential pre-stage of bronchiectasis. Although no previous studies focused on identifying pre-stages of bronchiectasis have been performed, an earlier study from our group identified mucus plugging as an indicator for bronchiectasis 6 years later [21]. Those results suggest a transition from mucus plugging to bronchiectasis, implying that optimising mucociliary clearance treatment is important to prevent bronchiectasis in CF. However, this hypothesis requires further investigation in a prospective CT study.

Development of bronchiectasis without clearly identifiable pre-stages 2 years earlier was commonly seen in this study. It is possible that the development of most bronchiectasis is an acute process and not caused by a slow continuous progressive transition. Therefore, we hypothesized that there are two possible phenotypes: one for rapid progression of bronchiectasis and one for more slowly developing bronchiectasis.

A sub-group analysis (rapid vs. slow progression) was performed to identify risk factors for the acute development of bronchiectasis in our relatively small single-centre cohort. This sub-group analysis showed only a borderline significant difference in baseline characteristics between the pancreatic status in the two groups. All patients in the rapid progression group were pancreatic insufficient, versus 80 % of the patients in the slow progression group. The importance of pancreatic status as a marker of disease severity has been observed in other studies [6, 22]. Although we could not identify an association between severe CFTR genotype and progression of bronchiectasis in our cohort aged 9–24 years, we must acknowledge our small sample size, which may have resulted in inadequate power to detect this association. Previous research by Mott et al. [6] did observe a significant association between genotype and bronchiectasis progression in children below the age of 6 years.

To gain further insight into the pathophysiology of bronchiectasis, it would be interesting to also include trapped air in the analyses, given that an association between trapped air and persistence or progression of bronchiectasis has recently been shown in young children with CF [6]. The most sensitive method to detect trapped air is an expiratory spirometer-controlled CT [23]. For our current study, we did not have sufficient spirometer-controlled expiratory CTs to include this analysis.

There are some limitations to this study. The longitudinal, retrospectively analysed data used in this study were collected from a single centre, which may reduce the generalizability of our results. Data were collected as part of the annual evaluation. During the study period our annual evaluation protocol changed to improve the structure of the follow-up schedule for routine chest CTs so that every child has a CT at the same age (2, 4, 6, 8, 10, 12, 14, 16 and 18 years) resulting in a larger time range between two CTs for some patients. CTs were performed on different CT scanners, due to the introduction of newer and quicker CT scanners. Furthermore, CT protocols changed during our study period to low-dose CT protocols and spirometer-controlled CT scanning, to reduce radiation and improve image quality [23]. We cannot exclude that the use of these different CT scanners and CT protocols may have reduced the sensitivity to detect pre stages of bronchiectasis. However, it is well recognized that the use of scoring systems is relatively insensitive to differences in CT scanners and CT protocols [24]. Our study is the first to identify ROIs and retrospectively assess the status of these regions in the years preceding bronchiectasis; this scoring strategy has not been used in other studies. Nevertheless, the kappa scores for the intra- and interobserver variability for the sub-scores were above 0.77 and 0.48, respectively. This suggests that our approach was sufficiently reproducible. Regarding the 301 ROIs with bronchiectasis in CT_baseline_, the two observers scored the same in all but four ROIs, indicating an excellent agreement between observers regarding baseline bronchiectasis. A disadvantage of our scoring method is that we may have missed changes such as subtle airway wall thickening, as scoring is considered a not very sensitive and reproducible method to quantify airway wall thickening [19]; in most CT-scoring studies the kappa for airway wall thickening is low. It is also very possible that when more sensitive automated image analysis tools are developed, subtle changes can be picked up and more pre-stages can be identified.

### Clinical implications

Our study shows the treacherous nature of CF lung disease in a cohort of patients who are receiving standard treatment as in the majority of cases bronchiectasis developed within 2 years without identifiable pre-stages. The only pre-stage that was identified in 17.7 % of cases of bronchiectasis was mucous impaction. Hence, this observation in a patient warrants close attention by the CF team to further improve mucociliary clearance. Furthermore, our results suggest that in some patients the development of bronchiectasis can occur rapidly. The identification of pre-stages of bronchiectasis early on might create a treatment opportunity before irreversible bronchiectasis occurs. Therefore, an annual CT may be considered in children with a rapid progression of bronchiectasis. Nevertheless, additional studies are needed to identify the risk factors leading to the sudden development of bronchiectasis. In future clinical intervention studies are needed that aim to prevent this ‘sudden’ development of bronchiectasis.

## Electronic supplementary material

Below is the link to the electronic supplementary material.

e-Figure 1.1 Different features of bronchiectasis. Figure 1.1a shows cylindric bronchiectasis. The upper arrow indicates the bronchiectasis and the lower arrow indicates the corresponding vessel. Figure 1.1b shows saccular bronchiectasis. The upper arrow indicates the corresponding vessel and the lower arrow indicates the saccular bronchiectasis. Figure 1.1c shows a lack of normal bronchial tapering for at least 2 cm, as indicated by the arrow. (JPG 29 kb)

High resolution image (TIFF 217 kb)

e-Figure 1.2 Illustration of scoring category: mucus plugging. The arrow in image 1.2a indicates a mucus filled bronchus. In Figure 1.2b it indicates the rosette pattern. The arrow in Figure 1.2c indicates the tree-in-bud sign and in Figure 1.2d it indicates a small mucous filled branching structure. (JPG 44 kb)

High resolution image (TIFF 316 kb)

e-Figure 1.3 Illustration of scoring category: airway wall thickening. In e-Figure 1.3a the left arrow indicates a vessel and the right arrow indicates airway wall thickening of a central airway. In e-Figure 1.3b the left arrow indicates a vessel and the right arrow indicates peripheral wall thickening. (JPG 18 kb)

High resolution image (TIFF 123 kb)

ESM 1 (DOCX 47 kb)

ESM 2 (DOCX 20 kb)

## Abbreviations

CF: Cystic fibrosis
CT: Computed tomography
FEV_1_: Forced expiratory volume in 1 s
FVC: Forced vital capacity
ROI: Region of interest
