# Supplementary figures and images for: The development of bronchiectasis on chest computed tomography in children with cystic fibrosis: can pre-stages be identified?
Source: Eur Radiol. 2016 Apr 23;26(12):4563–9. doi: 10.1007/s00330-016-4329-z (PMC5101271; doi:10.1007/s00330-016-4329-z)

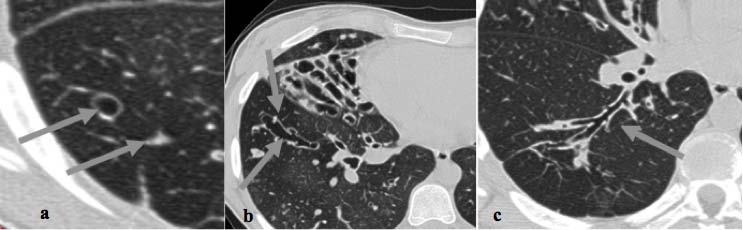

Supplement: Supplementary file 1 — Different features of bronchiectasis. Figure 1.1a shows cylindric bronchiectasis. The upper arrow indicates the bronchiectasis and the lower arrow indicates the corresponding vessel. Figure 1.1b shows saccular bronchiectasis. The upper arrow indicates the corresponding vessel and the lower arrow indicates the saccular bronchiectasis. Figure 1.1c shows a lack of normal bronchial tapering for at least 2 cm, as indicated by the arrow. (JPG 29 kb) [file 330_2016_4329_Fig4_ESM.jpg]

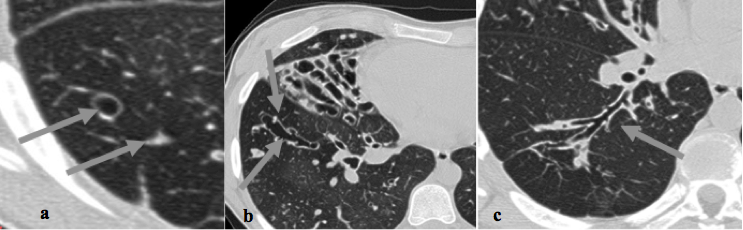

Supplement: Supplementary file 2 — High resolution image (TIFF 217 kb) [file 330_2016_4329_MOESM1_ESM.tiff]

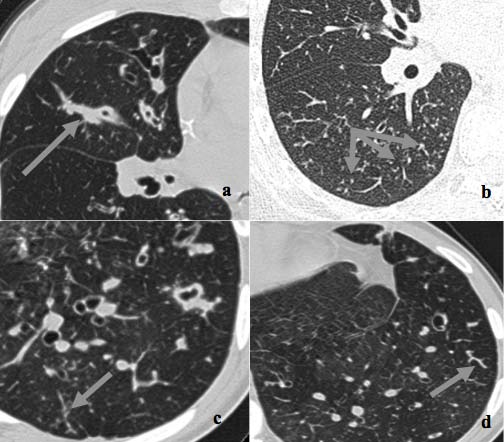

Supplement: Supplementary file 3 — Illustration of scoring category: mucus plugging. The arrow in image 1.2a indicates a mucus filled bronchus. In Figure 1.2b it indicates the rosette pattern. The arrow in Figure 1.2c indicates the tree-in-bud sign and in Figure 1.2d it indicates a small mucous filled branching structure. (JPG 44 kb) [file 330_2016_4329_Fig5_ESM.jpg]

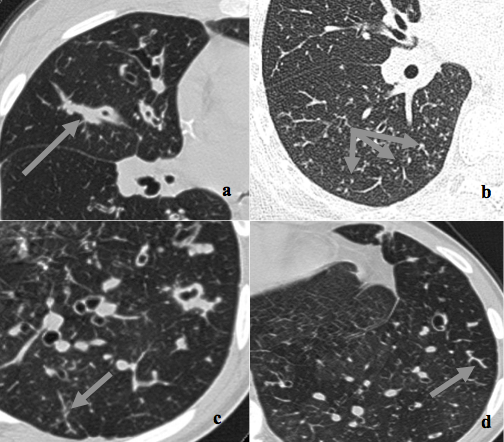

Supplement: Supplementary file 4 — High resolution image (TIFF 316 kb) [file 330_2016_4329_MOESM2_ESM.tiff]

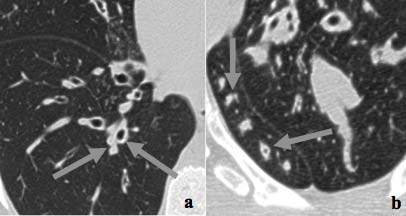

Supplement: Supplementary file 5 — Illustration of scoring category: airway wall thickening. In e-Figure 1.3a the left arrow indicates a vessel and the right arrow indicates airway wall thickening of a central airway. In e-Figure 1.3b the left arrow indicates a vessel and the right arrow indicates peripheral wall thickening. (JPG 18 kb) [file 330_2016_4329_Fig6_ESM.jpg]

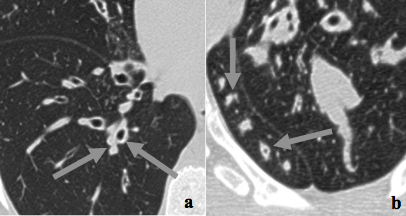

Supplement: Supplementary file 6 — High resolution image (TIFF 123 kb) [file 330_2016_4329_MOESM3_ESM.tiff]
