# Supplementary material for: The development of bronchiectasis on chest computed tomography in children with cystic fibrosis: can pre-stages be identified?
Source: Eur Radiol. 2016 Apr 23;26(12):4563–9. doi: 10.1007/s00330-016-4329-z (PMC5101271; doi:10.1007/s00330-016-4329-z)
Supplement: Supplementary file 7 — (DOCX 47 kb) [file 330_2016_4329_MOESM4_ESM.docx]

**e-Table 1.**  Scanning parameters for the different CT scanners.

|  | **Inspiratory** |  |  |  |  |  |
| --- | --- | --- | --- | --- | --- | --- |
| CT scanner | kV | kV | mAs | Rotation time | Collimation | Pitch |
| Emotion 6 | 110 (80^#^) | 110 | 20 CD4D | 0.6 | 6*2 | 1.50 |
| Definition Flash | 80 | 80 | 80 CD4D | 0.28 | 128*0.6 | 0.85 |
| Definition AS+ | 80 | 80 | 85 CD4D | 0.3 | 128*0.6 | 0.85 |
|  | **Expiratory** |  |  |  |  |  |
| Emotion 6 | 110 (80^#^) | 110 | 10 eff mAs | 0.6 | 6*2 | 1.50 |
| Definition Flash | 80 | 80 | 26 CD4D | 0.28 | 128*0-6 | 0.85 |
| Definition AS+ | 80 | 80 | 28 CD4D | 0.3 | 128*0.6 | 0.85 |

(^#^) kV voltages for children below 25 kg.

**e-Table 2.** Intra- and inter-observer agreement.


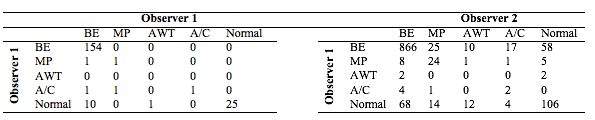


Left: intra-observer agreement (kappa 0.77) of observer 1 over a random subset of 195 regions of interest (ROI’s). Right: agreement between observer 1 and 2 over a random subset of 1230 regions of interest (kappa 0.48). In 866/1230 cases both observers agreed on bronchiectasis. Regarding the 301 ROI’s with bronchiectasis in CT_baseline_, the two observers score in all but 4 ROI’s similar, indicating a excellent agreement between observers regarding baseline bronchiectasis. BE: bronchiectasis, MP: mucus plugging, AWT: airway wall thickening, A/C: atelectasis or consolidation, Normal: no abnormalities.
