# Supplementary material for: The development of bronchiectasis on chest computed tomography in children with cystic fibrosis: can pre-stages be identified?
Source: Eur Radiol. 2016 Apr 23;26(12):4563–9. doi: 10.1007/s00330-016-4329-z (PMC5101271; doi:10.1007/s00330-016-4329-z)
Supplement: Supplementary file 8 — (DOCX 20 kb) [file 330_2016_4329_MOESM5_ESM.docx]

# ONLINE SUPPLEMENTARY MATERIAL

*CT acquisition*

CTs were performed on 6 different CT scanners, due to the introduction of new and faster scanners that allowed reducing radiation dose for our clinical chest CT protocol in the study period.

The included CTs (77.8%) were predominantly performed on the Emotion 6 (Siemens Emotion 6, Siemens Healthcare, Germany). In 2010, the Definition Flash scanner (SOMATOM Definition Flash, Siemens Healthcare, Germany) was introduced. In total 18.8% (33/176) of the included CTs were performed with the Definition Flash scanner (SOMATOM Definition Flash, Siemens Healthcare, Germany). The remaining 4% was performed on respectively the Definition AS+ (SOMATOM Definition AS+, Siemens Healthcare, Germany), the Definition Edge (SOMATOM Definition Edge, Siemens Healthcare, Germany), the Sensation 16 scanner (Siemens Sensation 16, Siemens Healthcare, Germany), and on the Biograph (Siemens Biograph, Siemens Healthcare, Germany). More details about the CT scanners are displayed in e-Table 1.

CTs were acquired using 3 mm thick images. Scanning parameters were 100kV and the tube current was modulated based on tissue.

For our spirometer controlled volumetric CT scanning protocol, patients were trained half an hour prior to the CT scan, to familiarise the patient with the spirometer and breathing manoeuvres in supine position. Patients were first instructed to breath normally to ensure a tidal breathing pattern. Subsequently for the inspiratory acquisition, the patient was instructed first to fully expire to residual volume, followed by maximal inspiration to total lung capacity. After the inspiratory manoeuvre the patient resumed tidal breathing. Next, the patient was instructed to fully inspire to total lung capacity, followed by a maximal expiration to residual volume. In 2007 spirometer controlled CT scanning was introduced, to optimize inspiratory and expiratory volume. In our study population, the majority (74.4%) of the included CT_baseline_ scans were spirometer controlled. However, if due to logistic reasons CT_baseline_ could not be spirometer controlled, then training for breath holds prior to scanning and instructions during scanning were given by the lung function technician.

*CT analysis*

We identified five mutually exclusive categories, according to the definitions of the well-validated CF-CT scoring system (18-20). The first category is the presence of bronchiectasis. Bronchiectasis was present if the bronchial lumen diameter is larger than the adjacent pulmonary artery outer diameter, or if there was a lack of tapering for at least 2 cm distal to a branching point (e-Figure 1.1). The second category is mucus plugging. Mucus plugging is defined as complete or incomplete filling of clearly identifiable bronchi, resulting in abnormal branching structures or centrilobular nodules in a rosette pattern or tree-in-bud sign (e-Figure 1.2). The third category is the presence of airway wall thickening, which is defined as the ratio between the bronchial wall thickness and the outer diameter of the adjacent pulmonary artery being more than 33% (e-Figure 1.3). The fourth is the presence of atelectasis or consolidation. The last category includes normal airways.

CTs were de-identified and randomized. One observer (observer 2: D.Z.) encircled all areas with bronchiectasis in the most recent volumetric inspiratory CT (CT_baseline_). Those areas were marked as a region of interest (ROI) using tools in our image analysis platform (**®**Myrian Onco XL, *Intrasense, France*). By using deformable image registration, the same areas were automatically identified in the previous CTs (CT_minus1;minus8_). All preceding ROIs were assessed by two experienced observers with respectively 1 and 2 years of experience (HO and DZ) and assigned to one of the 5 above-mentioned categories. The intra- and inter- observer agreement was calculated. More detailed information about the intra- and inter-observer variability is presented in e-Table 2. In case in a particular ROI more than one of the above mentioned categories was present, the following rating was applicable: bronchiectasis and mucus plugging were rated as bronchiectasis; bronchiectasis and airway wall thickening were rated as bronchiectasis; and if in our region of interest mucus plugging and airway wall thickening were present, this was rated as mucus plugging. ROIs were assessed in complete random order with respect to patients and order of CT-scans (_minus2 to minus8_). Therefore, the observers had no knowledge on the assigned category of the ROI’s in previous or later scans.
